# Role of ERK Pathway in the Pathogenesis of Atopic Dermatitis and Its Potential as a Therapeutic Target

**DOI:** 10.3390/ijms23073467

**Published:** 2022-03-23

**Authors:** Nahoko Zeze, Makiko Kido-Nakahara, Gaku Tsuji, Eriko Maehara, Yuki Sato, Sawako Sakai, Kei Fujishima, Akiko Hashimoto-Hachiya, Masutaka Furue, Takeshi Nakahara

**Affiliations:** 1 Department of Dermatology, Graduate School of Medical Sciences, Kyushu University, Fukuoka 812-8582, Japan; zeze.nahoko.235@s.kyushu-u.ac.jp (N.Z.); nakahara.makiko.107@m.kyushu-u.ac.jp (M.K.-N.); tsuji.gaku.893@m.kyushu-u.ac.jp (G.T.); eriko.maehara.655@s.kyushu-u.ac.jp (E.M.); sato.yuki.834@m.kyushu-u.ac.jp (Y.S.); sakai.sawako.400@m.kyushu-u.ac.jp (S.S.); fujishima.kei.855@m.kyushu-u.ac.jp (K.F.); hachiya.akiko.536@m.kyushu-u.ac.jp (A.H.-H.); furuemasutaka00@yahoo.co.jp (M.F.); 2 Research and Clinical Center for Yusho and Dioxin, Kyushu University, Maidashi 3-1-1, Fukuoka 812-8582, Japan

**Keywords:** atopic dermatitis, ERK pathway, filaggrin, TEWL, barrier function

## Abstract

Atopic dermatitis (AD) is an eczematous skin disorder characterized by type 2 inflammation, barrier disruption, and intense itch. In addition to type 2 cytokines, many other cytokines, such as interferon gamma (IFN-γ), interleukin 17 (IL-17), and interleukin 22 (IL-22), play roles in the pathogenesis of AD. It has been reported that the extracellular signal-regulated kinase (ERK) is downstream of such cytokines. However, the involvement of the ERK pathway in the pathogenesis of AD has not yet been investigated. We examined the expression of p-ERK in mouse and human AD skin. We also investigated the effects of the topical application of an ERK inhibitor on the dermatitis score, transepidermal water loss (TEWL), histological change, and expression of filaggrin, using an AD-like NC/Nga murine model. The effects of an ERK inhibitor on filaggrin expression in normal human epidermal keratinocytes (NHEKs) and on chemokine production from bone marrow-derived dendritic cells (BMDCs) were also evaluated. p-ERK was highly expressed in mouse and human AD skin. Topical application of an ERK inhibitor alleviated the clinical symptoms, histological changes, TEWL, and decrease in expression of filaggrin in the AD-like NC/Nga murine model. The ERK inhibitor also restored the IL-4 induced reduction in the expression of filaggrin in NHEK, and inhibited chemokine production from BMDC induced by IL-4. These results indicate that the ERK pathway is involved in the pathogenesis of AD, and suggest that the ERK pathway has potential as a therapeutic target for AD in the future.

## 1. Introduction

Atopic dermatitis (AD) is a common and heterogeneous eczematous skin disorder characterized by type 2 inflammation, barrier disruption, and chronic pruritus [1,2,3,4,5,6]. Frequent relapse with intense pruritus reduces the quality of life and decreases treatment satisfaction of afflicted patients [7,8]. Skin barrier dysfunction is associated with reduced production of terminal differentiation molecules, such as filaggrin [9,10,11,12]. Abnormal skin barrier integrity also causes increased colonization of microbes, such as *Staphylococcus aureus*, which further exacerbates Th2-deviated skin inflammation [1,13]. Recent therapeutic success with the anti-interleukin-4 (IL-4) receptor α antibody dupilumab suggests the pivotal role of IL-4 and IL-13 in the pathogenesis of AD because dupilumab interferes with both IL-4 and IL-13 signaling [14]. IL-4/IL-13 signaling downregulates the expression of filaggrin (FLG), loricrin (LOR), and involucrin (IVL) via signal transducer and activator of transcription 6 (STAT6) activation, impairing the epidermal terminal differentiation and barrier dysfunction [10,11]. IL-4/13 act primarily through the Janus kinase (JAK)-STAT pathway. IL-4/13 are also known to activate mitogen-activated protein kinases (MAPK) in various cell types. AD is diverse, and, in addition to Th2 cells, Th1, Th17, and Th22 cells are known to be involved in its pathogenesis [15]. It has also been reported that the MAPK pathway is downstream of the major cytokines produced by each of them, such as IFN-γ, IL-17, and IL-22 [16,17]. MAPK constitutes major signaling pathways from the cell surface to the nucleus in mammalian cells [18,19,20,21]. There are three main MAPK family members: extracellular signal-regulated kinase (ERK), c-Jun N-terminal kinases (JNK) and p38MAPK. MAPK signaling is altered in many diseases, including allergic disorders, and is one of the targets in drug development [18,19,21,22,23,24]. Among the pathways, the ERK pathway has been reported to be involved in the barrier function of the epidermis. Furthermore, we have shown that the expression of pruritogen endothelin-1 (ET-1) in the epidermis [25,26], migration of lymphocytes in response to thymus and activation-regulated chemokine(TARC) [27], and expression of IL-33 in the epidermis induced by IL-4 stimulation are mediated by the ERK pathway [28]. However, the expression of ERK in AD skin, its involvement in the pathogenesis of AD, and the therapeutic effects of inhibiting the ERK pathway have not been clarified. Therefore, in this study, we decided to analyze the role of ERK in the pathogenesis of AD and the therapeutic effects of its inhibitors.

## 2. Results

### 2.1. p-ERK Is Highly Expressed in Mouse and Human AD Skin

**Figure 1:**
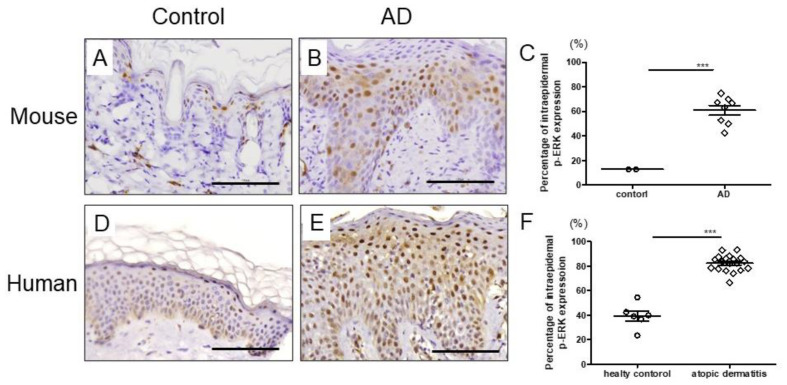
The expression of p-ERK in mouse and human skin. Immunohistochemical staining for p-ERK in normal (**A**,**D**) and AD (**B**,**E**) skin. Scale bar: 100 μm (**C**) Percentage of intraepidermal p-ERK expression of control (n = 2) and AD (n = 8) in mice. (**F**) Percentage of intraepidermal p-ERK expression of control (n = 6) and AD (n = 17) in human. All data are presented as mean ± standard error of the mean (S.E.M.). *** *p* < 0.001 Student’s unpaired two-tailed *t*-test.

We first investigated p-ERK expression in the skin of AD using skin samples from AD patients and mite antigen-induced AD mice to confirm if there is a link between AD and the ERK pathway. It is well known that mite antigen treatment clinically and histopathologically induces AD-like skin lesions [25]. Immunohistochemical analysis showed clear upregulation of p-ERK in the epidermis of AD mice compared with that in controls (Figure 1A–C). Enhanced expression of p-ERK was also evident in the epidermis of patients with AD compared with that in controls (Figure 1D–F), which suggests that the ERK pathway might play some role in the pathogenesis of AD. On the other hand, there was no difference in p-p38MAPK or p-JNK expression between AD patients and healthy controls (Figure S1). Based on these results, we decided to see if inhibition of the ERK pathway would improve the skin symptoms of AD using an AD mouse model.

### 2.2. Topical Application of ERK Inhibitor Alleviates the Clinical Symptoms of Mite Antigen-Induced AD in Mice

**Figure 2:**
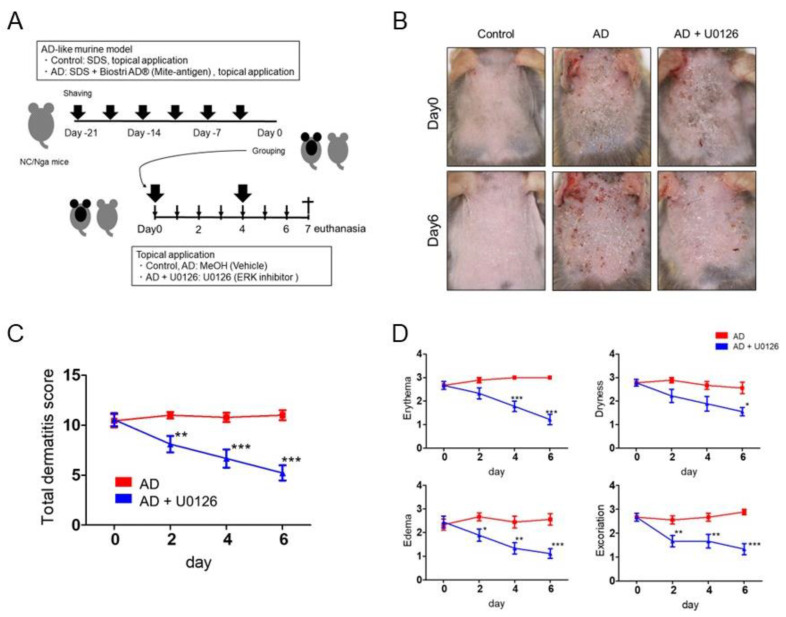
Topical application of ERK inhibitor alleviates the clinical symptoms of mite antigen-induced AD in NC/Nga mice. (**A**) Schema of induction of mite antigen-induced AD-like murine model. After 2 h treated with 4% SDS, mite antigen, *Dermatophagoides farinae* extract (Biostir AD^®^), was epicutaneously applied to the upper back and ear skin of mice twice a week for three weeks to develop AD. These mice were then topically treated with ERK inhibitor or vehicle for 7 days on the same site. (**B**) Macroscopic features of skin lesions of control, AD, and AD + U0126 groups on day 0 and 6. (**C**,**D**) Total dermatitis score (**C**) and all dermatitis scores such as erythema, dryness, edema, and excoriation (**D**) were assessed on day 0, 2, 4, and 6. All data are presented as mean ± S.E.M. (n = 9 per group). * *p* < 0.05, ** *p* < 0.01, *** *p* < 0.001 Student’s unpaired two-tailed *t*-test on each day.

Topical cutaneous application of mite antigen in this study induced more severe dermatitis in the AD group compared with the status in the control group (Figure 2A,B) [25,29]. Topical application of ERK inhibitor (U0126) significantly reduced clinical manifestations (Figure 2B) and total dermatitis scores (Figure 2C). ERK inhibitor decreased all dermatitis scores, such as erythema, dryness, edema, and skin excoriation quickly and significantly (Figure 2D). Specifically, ERK inhibitor significantly improved erythema from day 4 to 6, dryness from day 4 to 6, edema from day 4 to 6, and excoriation from day 2 to 6 (Figure 2D). No adverse events were observed with topical application of the ERK inhibitor.

### 2.3. Topical Application of ERK Inhibitor Improved the Histological Changes of Mite Antigen-Induced AD in Mice

**Figure 3:**
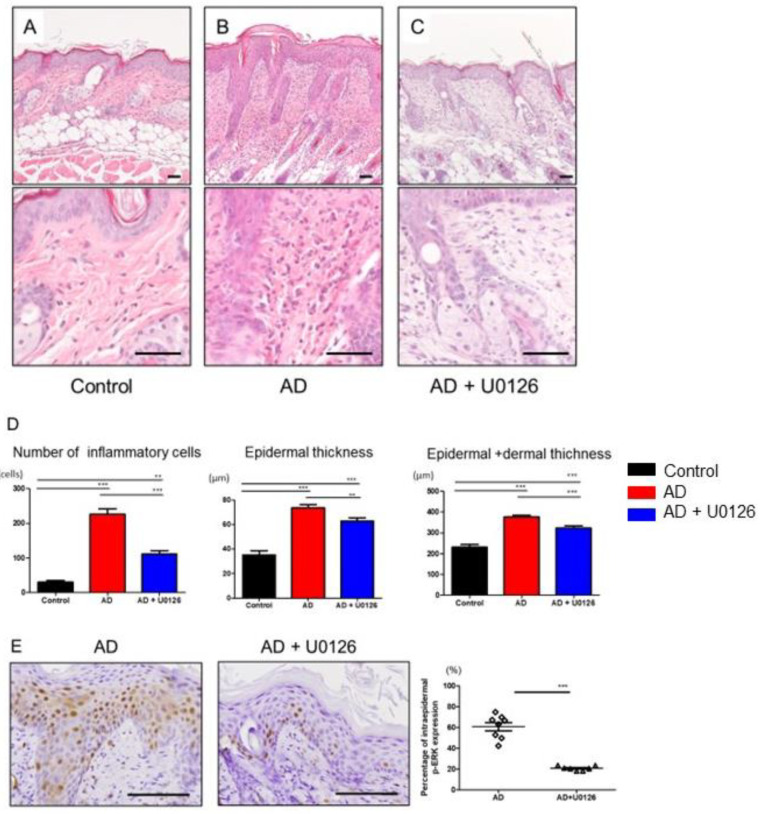
Topical application of ERK inhibitor improves the histological changes of mite antigen-induced AD in mice. Histological appearance (hematoxylin-eosin staining) of skin lesions of control (**A**), AD (**B**), and AD + U0126 (**C**) groups on day 7. Scale bar: 50 μm. (**D**) Number of total inflammatory cells in dermis on the dorsal skin of mice. Measurements of epidermal thickness and epidermal + dermal thickness ** *p* < 0.01, *** *p* < 0.001 one-way analysis of variance followed by Bonferroni’s multiple comparison test. (**E**) Immunohistochemical staining for intraepidermal p-ERK expression of skin lesions of AD and AD + U0126 groups. Scale bar: 100 μm., *** *p* < 0.001 Student’s unpaired two-tailed *t*-test on each day. All data are presented as mean ± S.E.M. (n = 3 control group, n = 8 AD group, n = 8 AD + U0126 group).

Histopathologically, AD is mainly characterized by epidermal and dermal hyperplasia and inflammatory cell infiltration [29]. Consistent with the clinical scores, histological analysis of skin samples on day 7 showed more severe epidermal hyperplasia and more intense inflammatory cell infiltration in AD mice than in control mice (Figure 3A,B). Topical ERK inhibitor treatment significantly improved the histological changes induced by mite antigen, attenuating the epidermal or epidermal and dermal hyperplasia, inflammatory cell infiltration, and eosinophils (Figure 3A–D and Figure S2). In addition, ERK inhibitor treatment decreased the expression of p-ERK in the epidermis of dermatitis while vehicle did not (Figure 3E).

### 2.4. Topical Application of ERK Inhibitor Improved TEWL and Restored FLG Expression in Mite Antigen-Induced AD in Mice

Next, to evaluate the effects of the topical ERK inhibitor on barrier dysfunction of AD, we analyzed TEWL and the expression of filaggrin in AD mice. TEWL is the slight transpiration of moisture through the epidermis and is widely used as an indicator of skin barrier function [30].

**Figure 4:**
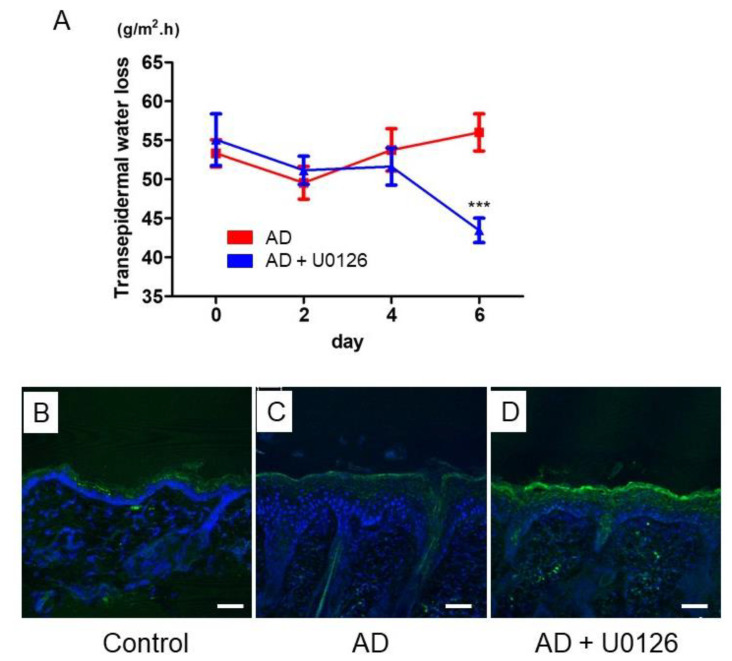
Topical application of ERK inhibitor improved TEWL and restored FLG expression in mite antigen-induced AD in mice. (**A**) TEWL was measured on the lesion by a Vapo Scan instrument on day 0, 2, 4, and 6. Data were presented as the average of five points repeated recordings. All data are presented as mean ± S.E.M. (n = 9 per group). *** *p* < 0.001 Student’s unpaired two-tailed t-test on each day. Immunohistochemical analysis of FLG protein (green) in the back skin in control (**B**), AD (**C**), AD + U0126 (**D**) groups on day 7. Nuclei were counterstained with DAPI (blue). Scale bar: 50 μm.

The group treated with topical ERK inhibitors showed significant improvement of TEWL on day 6 of application (Figure 4A). The expression of FLG in control mice was detectable in the granular layer of the epidermis (Figure 4B), but it was reduced in the AD mice (Figure 4C). However, topical ERK inhibitor recovered the expression of FLG in the epidermis of the AD mice (Figure 4D). These results suggested that topical ERK inhibitor improved barrier function by restoring filaggrin expression.

### 2.5. ERK Inhibitor Restored the Reduction in the Expression of Filaggrin in NHEK

**Figure 5:**
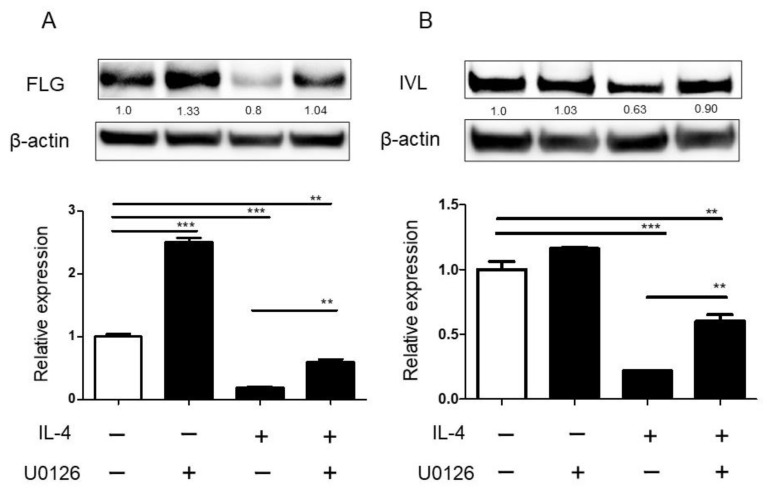
ERK inhibitor restored the reduction in the expression levels of filaggrin and involucrin in NHEK. NHEKs were treated with IL-4 (20 ng/mL) in the presence or absence of ERK inhibitor (1 µM) for 72 h (FLG) or 48 h (IVL) for qRT-PCR and for 96 h (FLG) or 72 h (IVL) for Western blotting analyses. FLG (**A**) and IVL (**B**) mRNA expression was analyzed by qRT-PCR. FLG (**A**) and IVL (**B**) protein expression was analyzed by Western blotting. All data are presented as mean ± S.E.M. (n = 3 per each group). ** *p* < 0.005, *** *p* < 0.001 one-way analysis of variance followed by Bonferroni’s multiple comparison test.

To evaluate the direct effects of ERK inhibitor on keratinocytes, we used human primary keratinocytes. When NHEKs were stimulated with IL-4, p-ERK were detected (Figure S3). In addition, IL-4 stimulation decreased the expression of filaggrin mRNA or involucrin mRNA, as previously reported (Figure 5A,B). ERK inhibitor significantly restored the reduction in the expression of filaggrin mRNA and protein in NHEK (Figure 5A). ERK inhibitor also partially, but significantly, restored the reduction in the expression of involucrin mRNA and protein in NHEK (Figure 5B).

### 2.6. ERK Inhibitor Inhibited Chemokine Production from BMDC Induced by IL-4

**Figure 6:**
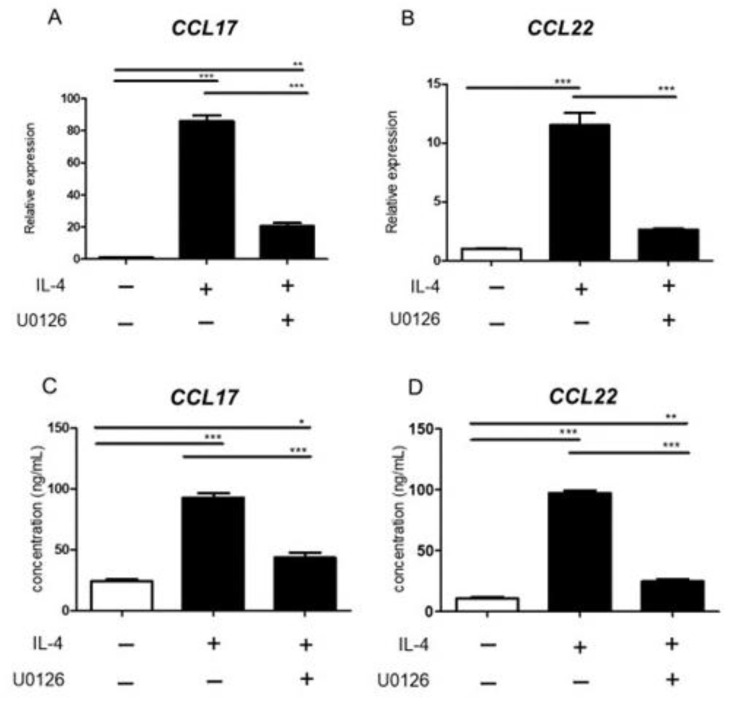
ERK inhibitor inhibited chemokine production from BMDC induced by IL-4. BMDCs were treated with IL-4 (20 ng/mL) in the presence or absence of ERK inhibitor. (**A**,**B**) CCL17 and CCL22 mRNA expression was analyzed by qRT-PCR. (**C**,**D**) CCL17 and CCL22 protein expression was analyzed by ELISA. All data are presented as mean ± S.E.M. (n = 3 per each group). * *p* < 0.05, ** *p* < 0.005, *** *p* < 0.001 one-way analysis of variance followed by Bonferroni’s multiple comparison test.

Chemokines from DCs play an important role in inflammatory cell infiltration in AD. It is well known that C-C motif chemokine 17 (CCL17) /TARC and CCL22/ macrophage-derived chemokine (MDC) are important in inflammation of AD because they cause Th2 cell migration. In histological analysis, ERK inhibitor decreased infiltration of inflammatory cells. So, we investigated the effects of ERK inhibitor on chemokine production from BMDCs. IL-4 stimulation also increased the mRNA and protein levels of CCL17 (Figure 6A,C) and CCL 22 (Figure 6B,D) in BMDCs. ERK inhibitor significantly inhibited the mRNA and protein levels of CCL17 (Figure 6A,C) and CCL 22 (Figure 6B,D) in BMDCs.

## 3. Discussion

The pathogenesis of AD is a combination of barrier dysfunction, inflammation, and intense itch [1,2,3,4,5,6], and its detailed pathogenesis is gradually becoming clearer. Accordingly, many new therapeutic agents have recently been developed and shown to be effective in many patients. However, not all skin rashes disappear, even with new systemic therapy, such as dupilumab or baricitinib [14,31,32], and it is important to combine it with topical therapy. Current topical medications include steroids, tacrolimus, and delgocitinib, but having other options may lead to better AD treatment. In addition, it would be useful to determine whether each topical medication specifically affects barrier function, inflammation, or itch in the pathogenesis of AD to help differentiate the use of topical medications.

In this study, we focused on the ERK pathway as a therapeutic target for AD. We have previously reported that the ERK pathway is involved in ET-1 regulation in the epidermis of AD [25,33]. ET-1 has been reported to be very important in the itch and chronicity of inflammation in AD [29]. We have also shown that the ERK pathway is involved in the migration of lymphocytes to TARC [27] and that the ERK pathway is important for IL-4-induced IL-33 expression in the epidermis [28]. TARC is an important biomarker in clinical practice and a very important chemokine for Th2 migration in lesions of AD [34,35]. IL-33 is known to cause inflammation and itch in AD [36]. These studies indicated that the ERK pathway in the epidermis and inflammatory cells may be involved in the pathogenesis of AD. Therefore, we analyzed whether p-ERK was actually expressed in AD skin, and found that it was strongly expressed mainly in the epidermis. In AD, the epidermal keratinocytes are important cells involved in both barrier function and inflammation. Topical application of an ERK inhibitor to AD mice revealed improvements in skin findings, pathological findings, and barrier function. Since filaggrin expression in epidermis is very important in barrier function, we evaluated filaggrin expression and found that the expression decreased in AD and was restored by topical ERK inhibitor. In other words, ERK inhibitor may improve the skin rash of AD by acting on the epidermis and restoring filaggrin expression. This mechanism of action was supported by validation using NHEK. ERK inhibitor restored the IL-4-induced decrease in filaggrin expression in NHEK. There are many reports on the involvement of ERK pathway and skin barrier function which are consistent with our results [37,38]. It has been reported that the downregulation of filaggrin expression by IL-33 and TSLP is mediated by the ERK pathway [39]. It has also been reported that the ERK pathway is involved in IL-33-induced reduction in claudin-1 (CLDN1) expression [28]. In addition to filaggrin, tight junctions (TJs) have important roles in skin barrier function. The TJ protein CLDN1 is decreased in AD [40].

In addition, because the pathological infiltration of inflammatory cells decreased, TARC production, which is particularly important for Th2 migration in AD, was examined using BMDCs, and ERK inhibitor suppressed the IL-4-induced increase in TARC production from BMDCs. Taken together with our previous report, inhibition of the ERK pathway may inhibit inflammatory cell infiltration into the lesional skin of AD by suppressing both chemokine production from DCs and chemokine-induced migration of lymphocytes [27].

A limitation of this study is that the duration of topical application of ERK inhibitor should be longer to maximize the effect of ERK inhibitor, but it was not possible because skin inflammation naturally tended to disappear even in the control group as the period of time increased. Another limitation of this study is that we used only U0126 as the ERK inhibitor. Although the importance of the ERK pathway can be fully examined using several inhibitors, the specificity of U0126 as an ERK inhibitor has already been well validated.

In conclusion, ERK expression was observed in AD skin and may play some role in the pathogenesis of AD. In fact, topical ERK inhibitors improved skin barrier function, suppressed inflammatory cell infiltration, and improved dermatitis. These results suggest that ERK inhibitors may be a novel therapeutic target in AD for the future.

## 4. Materials and Methods

### 4.1. Reagents and Antibodies

U0126 was purchased from Cell Signaling Technology (Danvers, MA, USA). The molecular weight of U0126 is 380 g/mol. The U0126 was dissolved in dimethyl sulfoxide (DMSO; Sigma-Aldrich, St. Louis, MO, USA) and stored at −30 °C until use for experiments with normal human epidermal keratinocytes (NHEKs) and bone marrow-derived dendritic cells (BMDCs), and in methanol 13 mM (0.5%) for NC/Nga mouse experiments. Human recombinant IL-4 was obtained from PeproTech (Rocky Hill, Cranbury, NJ, USA). Anti-filaggrin mouse monoclonal antibody (Santa Cruz Biotechnology, Dallas, TX, USA), anti-involucrin mouse monoclonal antibody (Abcam, Cambridge, UK), anti-phosphorylated-p44/42 MAPK (Erk1/2) rabbit monoclonal antibody (Cell Signaling Technology, Danvers, MA, USA), anti-p44/42 MAPK (Erk1/2) rabbit monoclonal antibody (Cell Signaling Technology, Danvers, MA, USA), and anti-β-actin mouse monoclonal antibody (Cell Signaling Technology, Danvers, MA, USA) were used for Western blotting. Anti-phosphorylation-p44/42 MAPK (Erk1/2) rabbit monoclonal antibody (Cell Signaling Technology, Danvers, MA, USA), anti- filaggrin rabbit polyclonal antibody (BioLegend, Covance, Princeton, NJ, USA) were used for immunohistochemical staining.

### 4.2. Mice

Female NC/Nga mice aged 9–10 weeks were used for the experiment on AD mouse models. These were purchased from SLC Japan (Hamamatsu, Japan). Female C57BL/6 mice aged 6 weeks were used for the experiment on BMDCs. These were purchased from Kyudo (Tosu, Japan).

The mice were maintained under a 12-h light/dark cycle under specific pathogen-free conditions. The animal experiments in this study were approved by the Animal Care and Use Committee of Kyushu University School of Medicine.

### 4.3. Induction of Mite Antigen-Induced AD-like Murine Model 

Mice were anesthetized with sevoflurane and the hair on the upper back was shaved with clippers. After 2 h treated with 150 µL of 4% SDS for disruption of barrier, 100 mg of mite-antigen, *Dermatophagoides farina extract* (Biosir AD^®^, Biostir Inc., Osaka, Japan) was epicutaneously applied to the upper back and ear skin of mice twice a week for three weeks to develop AD. The mice were then divided into the following three groups: Control group, AD group, and AD + U0126 group at day 0. Mice were then topically treated with 120 µL of 5% U0126 or vehicle for 7 days on the same site and were euthanized at day 7.

### 4.4. Evaluation of Skin Lesions

The clinical severity of dermatitis was assessed on day 0, 2, 4, and 6 after the start of treatment. The development of erythema/hemorrhage, scaling/dryness, edema, and skin excoriation/erosion was scored as 0 (none), 1 (mild), 2 (moderate), or 3 (severe). The total dermatitis score was the sum of these scores.

### 4.5. Transepidermal Water Loss (TEWL)

TEWL was measured on the skin lesion by a Vapo Scan AS-VT100RS machine (ASCH Japan Co., Ltd., Tokyo, Japan). Measurement of TEWL was performed at room temperature (23–26 °C) and room humidity (40–55%) in the animal facility center of Kyushu University.

### 4.6. Histological Examination and Immunohistochemical Staining

Portions of the dorsal skin of mice were fixed in 10% neutral formalin, embedded in paraffin, and OCT compounded in liquid nitrogen. Fixed paraffin samples were sectioned at 3 μm and compounded samples were sectioned at 5 µm. Formalin-fixed and paraffin-embedded tissues of human skin were fixed in 4% phosphate-buffered formalin, embedded in paraffin. Sections were deparaffinized, rehydrated, and stained with hematoxylin and eosin. Sections for anti-phosphorylation-p44/42 MAPK (Erk1/2) staining were deparaffinized, rehydrated, and pressure-cooked in 10 mM sodium citrate buffer (Histofine PRO II) at pH 6.0. Sections were immersed in 3% hydrogen peroxide solution for 30 min. After blocking with 10% goat serum for 30 min, sections were incubated with anti-phosphorylation-p44/42 MAPK (Erk1/2) diluted in 1:1000 at 4 °C overnight. Subsequently, the sections were incubated with secondary antibody Simplestain mouse MAX-PO (R) (Nichirei biosciences INC, Tokyo, Japan) for 30 min. Immunoreactions were detected using a Liquid DAB+ Substrate Chromogen System (Nichirei biosciences INC, Tokyo, Japan). Frozen sections were immersed in 3% hydrogen peroxide solution in methanol to block endogenous peroxidase activity and incubated with anti-FLG antibody at a dilution of 1:1000 for 1 h at room temperature. The sections were incubated with secondary antibody FITC goat anti-rabbit IgG (Abcam, Cambridge, UK) diluted 1:1000 for 30 min at room temperature under light shielding and mounted with mounting medium with DAPI (Vector Laboratories, Burlingame, CA, USA). These were observed by confocal microscopy (TCS SP8; Leica, Tokyo, Japan).

To assess the number of inflammatory cells in the dermis, three high-magnification fields per skin section from each mouse were randomly selected and the average number of stained cells counted (n = 3 control group, n = 8 AD group, n = 8 AD + U0126 groups). For quantification of p-ERK expression, three high magnification fields were randomly selected and the percentage of stained cell nuclei in the epidermis was counted (n = 2 control group, n = 8 AD group, n = 8 AD + U0126 groups). In measuring epidermal thickness and epidermis + dermis thickness, measurements were taken at five random locations with a low magnification field of view and averaged.

### 4.7. Cell Culture

Normal human epidermal keratinocytes (NHEKs), obtained from Lonza (Basel, Switzerland), were grown in culture dishes at 37 °C in 5% carbon dioxide. The NHEKs were cultured in serum-free keratinocyte growth medium (Lonza) supplemented with bovine pituitary extract, recombinant epidermal growth factor, insulin, hydrocortisone, transferrin and epinephrine. Culture medium was replaced every 2 days. Near confluence (70–90%), cells were disaggregated with 0.25 mg/mL trypsin/0.01% ethylenediaminetetraacetic acid and subcultured. Second- to fourth-passage NHEKs were used in all experiments. Normal human epidermal keratinocytes (1 × 10^5^) were seeded in 24-well culture plates and allowed to attach for 24 h. The cells were pre-treated with or without 1 μM U0126 for 1 h and subsequently treated with or without 20 ng/mL IL-4 (PeproTech, Cranbury, NJ, USA) for 24 h.

### 4.8. Generation of Bone Marrow-Derived DCs (BMDCs) 

Bone marrow (BM) cells freshly isolated from C57BL/6 mice were cultured in RPMI 1640 medium (Sigma-Aldrich Co. LLC, St. Louis, MO, USA) supplemented with 10% FBS (Capricorn Scientific GmbH, Ebsdorfergrund, Germany), 10 mmol/L, 4-(2-hydroxyethyl)-1-piperazineethanesulfonic acid (HEPES) (Thermo Fisher Scientific, Waltham, MA, USA), 1% minimum essential medium non-essential amino acids (MEM NEAA) 10 mL/L, (Thermo Fisher Scientific), 1 mmol/L sodium pyruvate (Thermo Fisher Scientific), 50 nmol/L β-mercaptoethanol (Nakalai Tesque, INC, Kyoto Japan), penicillin-streptomycin-glutamine (100 U/mL penicillin, 100 µg/mL streptomycin and 29.2 mg/mL glutamine) (Thermo Fisher Scientific, Waltham, MA, USA), in the presence of GM-CSF (10 ng/mL) (PeproTech, Cranbury, NJ, USA). Medium was refreshed and GM-CSF was added, twice in 8 days. On day 10, non-adherent cells were harvested. These cells were purified immunomagnetically by two or three rounds of positive selection with CD11c (N418) MicroBeads (Miltenyi Biotec B.V. & Co., KG, Bergisch Gladbach, Germany) [41]. Purified BMDCs were seeded in 12-well or 24-well dishes, pre-treated with or without U0126 for 1h, and then subsequently treated with or without IL-4 (Peprotech) for 24 h. Culture supernatants were collected at 24 h and analyzed by ELISA. Cells were also collected for PCR analysis and western blot analysis as described below.

### 4.9. Real-Time Quantitative Reverse Transcriptase Polymerase Chain Reaction (qRT-PCR)

Total RNA was extracted from NHEKs using the RNeasy Plus Micro kit (QIAGEN, Hilden, Germany) to eliminate contaminating genomic DNA. Total RNA was extracted from BMDCs using the RNeasy Mini kit (QIAGEN, Hilden, Germany). Reverse transcription was performed using PrimeScript RT reagent kit (Takara Bio, Shiga, Japan). qRT-PCR was conducted on a CFX Connect Real-time System (Bio-Rad, Hercules, CA, USA) using TB Green Premix Ex TaqⅡ (Takara Bio). Amplification was initiated at 95 °C for 30 s. as the first step, followed by 40 cycles of qRT-PCR at 95 °C for 5 s and at 60 °C for 20 s. mRNA expression was measured in quadruplicate and normalized to the β-actin expression level. The sequences of primer pairs are shown in Table 1.

### 4.10. Western Blotting

Cells were incubated for 10 min in lysis buffer following the method described in Chamcheu J C et al. [42] with slight modification for the filaggrin sample. The protein concentration in the lysate was measured using a BCA Protein Assay Kit (Thermo Fisher Scientific, Rockford, IL, USA). Equal amounts of protein (20 or 30 μg) were dissolved in NuPAGE LDS sample buffer (Thermo Fisher Scientific) and a 10% sample reducing agent (Thermo Fisher Scientific). The lysates were boiled at 70 °C for 10 min and then loaded into and subjected to electrophoresis in NuPAGE 4–12% Bis-Tris gels (Thermo Fisher Scientific) at 200 V for 30 min. The proteins were then transferred onto polyvinylidene difluoride membranes (Thermo Fisher Scientific), which were blocked with WesternBreeze Blocker/Diluent (Thermo Fisher Scientific). The membranes were then probed with anti-phosphorylated-p44/42 MAPK (Erk1/2) antibody, anti-p44/42 MAPK (Erk1/2) antibody, anti-filaggrin antibody, anti-involucrin antibody, anti-β-actin antibody for 30min or overnight at 4 °C. Horseradish peroxidase-conjugated anti-rabbit or anti-mouse IgG antibodies (Cell Signaling Technology) served as secondary antibodies. The visualization of protein bands was accomplished with the SuperSignal West Pico Chemiluminescent Substrate (Thermo Fisher Scientific) using the ChemiDoc touch imaging system (Bio-Rad). The protein was measured by ImageJ (National Institutes of Health, Bethesda, MD, USA).

### 4.11. ELISA

Each culture supernatant was measured using murine CCL17/TARC and CCL22/MDC ELISA Kits (R&D Systems, Inc., Minneapolis, MN, USA), in accordance with the manufacturers’ protocols. Optical density was measured using a DTX 800 Multimode Detector (Beckman Coulter Inc., Brea, CA, USA).

### 4.12. Tissue Sample

Samples were obtained from the Department of Dermatology, Kyushu University. Six patients had healthy skin and seventeen patients had atopic dermatitis. This study was approved by the Institutional Ethics Committee of Kyushu University (number 2019-509).

### 4.13. Statistical Analysis

Statistical analysis was performed using GraphPad Prism Version 5 (GraphPad Software). All data are presented as mean ± S.E.M. The significance of differences between groups was assessed using Student’s unpaired two-tailed *t*-test or one-way analysis of variance, followed by Bonferroni’s multiple comparison test. *p*-value of <0.05 was considered statistically significant.

## 5. Conclusions

p-ERK expression was observed in AD skin and may play some role in the pathogenesis of AD. Topical ERK inhibitors improved skin barrier function, suppressed inflammatory cell infiltration, and alleviated dermatitis. These results suggest that the ERK pathway has potential as a therapeutic target for AD in the future.

## Figures and Tables

**Table 1:** The sequences of primer.

Gene			Sequence (5′to 3′)
human	β-actin	forward	ATTGCCGACAGGATGCAGA
		reverse	GAGTACTTGCGCTCAGGAGGA
human	Filaggrin	forward	CATGGCAGCTATGGTAGTGCAGA
		reverse	ACCAAACGCACTTGCTTTACAGA
human	Involucrin	forward	TAACCACCCGCAGTGTCCAG
		reverse	ACAGATGAGACGGGCCACCTA
mouse	β-actin	forward	GGCTGTATTCCCCTCCATCG
		reverse	CCAGTTGGTAACAATGCCATGT
mouse	CCL-17	forward	AGGTCACTTCAGATGCTGCTC
		reverse	ACTCTCGGCCTACATTGGTG
mouse	CCL-22	forward	GACACCTGACGAGGACACA
		reverse	GCAGAGGGTGACGGATGTAG

## Data Availability

Not applicable.

## Supplementary Materials

The following supporting information can be downloaded at: https://www.mdpi.com/article/10.3390/ijms23073467/s1.

## Abbreviations

AD	atopic dermatitis
IFN-γ	interferon gamma
IL-	interleukin
MAPK	mitogen-activated protein kinases
STAT	signal transducer and activator of transcription
JAK	Janus kinase
ERK	extracellular signal-regulated kinase
JNK	c-Jun N-terminal kinases
ET	endothelin
TARC	thymus and activation-regulated chemokine
NHEK	normal human epidermal keratinocytes
BMDC	bone marrow-derived dendritic cells
FLG	filaggrin
LOR	loricrin
IVL	involucrin
Th	T helper cell
TEWL	transepidermal water loss
CCL	C-C motif chemokine
MDC	macrophage-derived chemokine
CLDN	claudin
TJs	tight junctions

## References

[1] Brunner P.M., Leung D.Y.M., Guttman-Yassky E. (2018). Immunologic, microbial, and epithelial interactions in atopic dermatitis. Ann. Allergy Asthma Immunol..

[2] Furue M., Chiba T., Tsuji G., Ulzii D., Kido-Nakahara M., Nakahara T., Kadono T. (2017). Atopic dermatitis: Immune deviation, barrier dysfunction, IgE autoreactivity and new therapies. Allergol. Int..

[3] Furue M., Ulzii D., Vu Y.H., Tsuji G., Kido-Nakahara M., Nakahara T. (2019). Pathogenesis of Atopic Dermatitis: Current Paradigm. Iran. J. Immunol..

[4] Guttman-Yassky E., Krueger J.G. (2017). Atopic dermatitis and psoriasis: Two different immune diseases or one spectrum?. Curr. Opin. Immunol..

[5] Nakahara T., Kido-Nakahara M., Tsuji G., Furue M. (2021). Basics and recent advances in the pathophysiology of atopic dermatitis. J. Dermatol..

[6] Kabashima K. (2013). New concept of the pathogenesis of atopic dermatitis: Interplay among the barrier, allergy, and pruritus as a trinity. J. Dermatol. Sci..

[7] McKenzie C., Silverberg J.I. (2019). The prevalence and persistence of atopic dermatitis in urban United States children. Ann. Allergy Asthma Immunol..

[8] Nakahara T., Fujita H., Arima K., Taguchi Y., Motoyama S., Furue M. (2019). Treatment satisfaction in atopic dermatitis relates to patient-reported severity: A cross-sectional study. Allergy.

[9] Drislane C., Irvine A.D. (2020). The role of filaggrin in atopic dermatitis and allergic disease. Ann. Allergy Asthma Immunol..

[10] van den Bogaard E.H., Bergboer J.G., Vonk-Bergers M., van Vlijmen-Willems I.M., Hato S.V., van der Valk P.G., Schröder J.M., Joosten I., Zeeuwen P.L., Schalkwijk J. (2013). Coal tar induces AHR-dependent skin barrier repair in atopic dermatitis. J. Clin. Investig..

[11] Tsuji G., Hashimoto-Hachiya A., Kiyomatsu-Oda M., Takemura M., Ohno F., Ito T., Morino-Koga S., Mitoma C., Nakahara T., Uchi H. (2017). Aryl hydrocarbon receptor activation restores filaggrin expression via OVOL1 in atopic dermatitis. Cell Death Dis..

[12] Tsuji G., Ito T., Chiba T., Mitoma C., Nakahara T., Uchi H., Furue M. (2018). The role of the OVOL1-OVOL2 axis in normal and diseased human skin. J. Dermatol. Sci..

[13] Han H., Roan F., Ziegler S.F. (2017). The atopic march: Current insights into skin barrier dysfunction and epithelial cell-derived cytokines. Immunol. Rev..

[14] Simpson E.L., Bieber T., Guttman-Yassky E., Beck L.A., Blauvelt A., Cork M.J., Silverberg J.I., Deleuran M., Kataoka Y., Lacour J.P. (2016). Two Phase 3 Trials of Dupilumab versus Placebo in Atopic Dermatitis. N. Engl. J. Med..

[15] Czarnowicki T., He H., Krueger J.G., Guttman-Yassky E. (2019). Atopic dermatitis endotypes and implications for targeted therapeutics. J. Allergy Clin. Immunol..

[16] Meephansan J., Komine M., Tsuda H., Karakawa M., Tominaga S., Ohtsuki M. (2013). Expression of IL-33 in the epidermis: The mechanism of induction by IL-17. J. Dermatol. Sci..

[17] Lejeune D., Dumoutier L., Constantinescu S., Kruijer W., Schuringa J.J., Renauld J.-C. (2002). Interleukin-22 (IL-22) Activates the JAK/STAT, ERK, JNK, and p38 MAP Kinase Pathways in a Rat Hepatoma Cell Line. J. Biol. Chem..

[18] Kim E.K., Choi E.J. (2010). Pathological roles of MAPK signaling pathways in human diseases. Biochim. Biophys. Acta (BBA)-Mol. Basis Dis..

[19] Lee S., Rauch J., Kolch W. (2020). Targeting MAPK Signaling in Cancer: Mechanisms of Drug Resistance and Sensitivity. Int. J. Mol. Sci..

[20] Morrison D.K. (2012). MAP kinase pathways. Cold Spring Harb. Perspect. Biol..

[21] Semba T., Sammons R., Wang X., Xie X., Dalby K.N., Ueno N.T. (2020). JNK Signaling in Stem Cell Self-Renewal and Differentiation. Int. J. Mol. Sci..

[22] Donohoe F., Wilkinson M., Baxter E., Brennan D.J. (2020). Mitogen-Activated Protein Kinase (MAPK) and Obesity-Related Cancer. Int. J. Mol. Sci..

[23] Guo Y.J., Pan W.W., Liu S.B., Shen Z.F., Xu Y., Hu L.L. (2020). ERK/MAPK signalling pathway and tumorigenesis. Exp. Ther. Med..

[24] Kumar S., Principe D.R., Singh S.K., Viswakarma N., Sondarva G., Rana B., Rana A. (2020). Mitogen-Activated Protein Kinase Inhibitors and T-Cell-Dependent Immunotherapy in Cancer. Pharmaceuticals.

[25] Aktar M.K., Kido-Nakahara M., Furue M., Nakahara T. (2015). Mutual upregulation of endothelin-1 and IL-25 in atopic dermatitis. Allergy.

[26] Nakahara T., Kido-Nakahara M., Ohno F., Ulzii D., Chiba T., Tsuji G., Furue M. (2018). The pruritogenic mediator endothelin-1 shifts the dendritic cell-T-cell response toward Th17/Th1 polarization. Allergy.

[27] Moroi Y., Yu B., Urabe K., Koga T., Nakahara T., Dainichi T., Uchi H., Furue M. (2004). Effects of MAPK inhibitors on CCR4-mediated chemotaxis against thymus and activation-regulated chemokine (TARC/CCL17). J. Dermatol. Sci..

[28] Ryu W.I., Lee H., Bae H.C., Jeon J., Ryu H.J., Kim J., Imai Y., Yamanishi K. (2018). IL-33 down-regulates CLDN1 expression through the ERK/STAT3 pathway in keratinocytes. J. Dermatol. Sci..

[29] Kido-Nakahara M., Wang B., Ohno F., Tsuji G., Ulzii D., Takemura M., Furue M., Nakahara T. (2021). Inhibition of mite-induced dermatitis, pruritus, and nerve sprouting in mice by the endothelin receptor antagonist bosentan. Allergy.

[30] Alexander H., Brown S., Danby S., Flohr C. (2018). Research Techniques Made Simple: Transepidermal Water Loss Measurement as a Research Tool. J. Investig. Dermatol..

[31] Nakahara T., Izuhara K., Onozuka D., Nunomura S., Tamagawa-Mineoka R., Masuda K., Ichiyama S., Saeki H., Kabata Y., Abe R. (2020). Exploration of biomarkers to predict clinical improvement of atopic dermatitis in patients treated with dupilumab: A study protocol. Medicine.

[32] Guttman-Yassky E., Silverberg J.I., Nemoto O., Forman S.B., Wilke A., Prescilla R., de la Peña A., Nunes F.P., Janes J., Gamalo M. (2019). Baricitinib in adult patients with moderate-to-severe atopic dermatitis: A phase 2 parallel, double-blinded, randomized placebo-controlled multiple-dose study. J. Am. Acad. Dermatol..

[33] Kido-Nakahara M., Buddenkotte J., Kempkes C., Ikoma A., Cevikbas F., Akiyama T., Nunes F., Seeliger S., Hasdemir B., Mess C. (2014). Neural peptidase endothelin-converting enzyme 1 regulates endothelin 1–induced pruritus. J. Clin. Investig..

[34] Kakinuma T., Nakamura K., Wakugawa M., Mitsui H., Tada Y., Saeki H., Torii H., Asahina A., Onai N., Matsushima K. (2001). Thymus and activation-regulated chemokine in atopic dermatitis: Serum thymus and activation-regulated chemokine level is closely related with disease activity. J. Allergy Clin. Immunol..

[35] Kataoka Y. (2014). Thymus and activation-regulated chemokine as a clinical biomarker in atopic dermatitis. J. Dermatol..

[36] Imai Y., Yasuda K., Sakaguchi Y., Haneda T., Mizutani H., Yoshimoto T., Nakanishi K., Yamanishi K. (2013). Skin-specific expression of IL-33 activates group 2 innate lymphoid cells and elicits atopic dermatitis-like inflammation in mice. Proc. Natl. Acad. Sci. USA.

[37] Meng X., Qiu L., Song H., Dang N. (2018). MAPK Pathway Involved in Epidermal Terminal Differentiation of Normal Human Epidermal Keratinocytes. Open Med..

[38] Cursons J., Gao J., Hurley D.G., Print C.G., Dunbar P.R., Jacobs M.D., Crampin E.J. (2015). Regulation of ERK-MAPK signaling in human epidermis. BMC Syst. Biol..

[39] Ryu W.-I., Lee H., Kim J.H., Bae H.C., Ryu H.J., Son S.W. (2015). IL-33 induces Egr-1-dependent TSLP expression via the MAPK pathways in human keratinocytes. Exp. Dermatol..

[40] De Benedetto A., Rafaels N.M., McGirt L.Y., Ivanov A.I., Georas S.N., Cheadle C., Berger A.E., Zhang K., Vidyasagar S., Yoshida T. (2011). Tight junction defects in patients with atopic dermatitis. J. Allergy Clin. Immunol..

[41] Nakahara T., Oba J., Shimomura C., Kido-Nakahara M., Furue M. (2016). Early Tumor-Infiltrating Dendritic Cells Change their Characteristics Drastically in Association with Murine Melanoma Progression. J. Investig. Dermatol..

[42] Chamcheu J.C., Esnault S., Adhami V.M., Noll A.L., Banang-Mbeumi S., Roy T., Singh S.S., Huang S., Kousoulas K.G., Mukhtar H. (2019). Fisetin, a 3,7,3’,4’-Tetrahydroxyflavone Inhibits the PI3K/Akt/mTOR and MAPK Pathways and Ameliorates Psoriasis Pathology in 2D and 3D Organotypic Human Inflammatory Skin Models. Cells.

