# Supplementary material for: Role of ERK Pathway in the Pathogenesis of Atopic Dermatitis and Its Potential as a Therapeutic Target
Source: Int J Mol Sci. 2022 Mar 23;23(7):3467. doi: 10.3390/ijms23073467 (PMC8999015; doi:10.3390/ijms23073467)
Supplement: Supplementary file 1 [file ijms-23-03467-s001.zip › ijms-1565424-supplementary.pdf]

Supplementary Figure

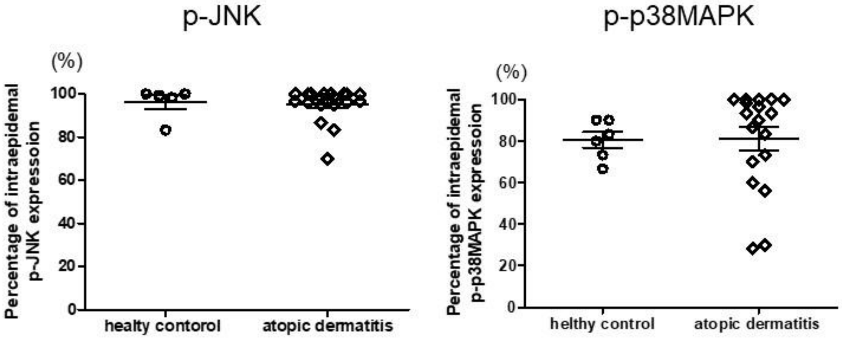

Figure S1. The expression of p-JNK, p-p38MAPK in human AD skin.

Percentage of p-JNK, p-p38MAPK expression of control (n=6) and AD (n=17) skin in human. All data are presented as mean ± standard error of the mean (S.E.M.). (Student's unpaired two-tailed t-test)

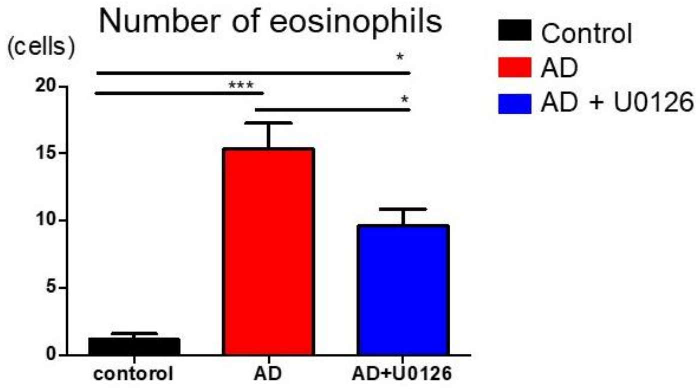

Figure S2. Number of eosinophils in dermis of the dorsal skin of mice.
All data are presented as mean ± S.E.M. (n=3 control group, n=8 AD group, n=8 AD + U0126 group). *P < 0.05, ***P < 0.001 (one-way analysis of variance followed by Bonferroni's multiple comparison test)

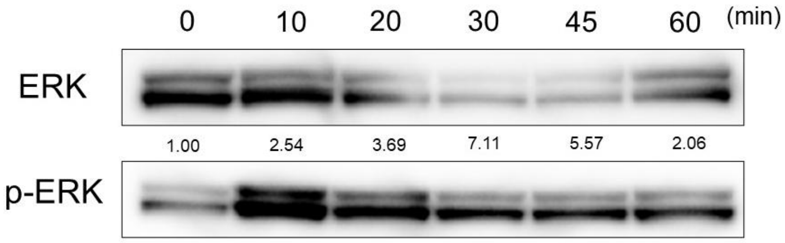

Figure S3. Phosphorylation of ERK in NHEK

NHEKs were stimulated with IL-4 (20 ng/mL) for 10, 20, 30, 45 and 60 min and then total protein of the NHEKs was extracted to measure the induction of phosphorylation of ERK by western blotting.
